# Association between vitamin D deficiency and lipid profiles in overweight and obese adults: a systematic review and meta-analysis

**DOI:** 10.1186/s12889-023-16447-4

**Published:** 2023-08-29

**Authors:** Xiao Huang, Yan Yang, Yingling Jiang, Zhiguang Zhou, Jingjing Zhang

**Affiliations:** 1 Metabolic Syndrome Research Center, Key Laboratory of Diabetes Immunology (Central South University), Ministry of Education, Department of Metabolism and Endocrinology, National Clinical Research Center for Metabolic Diseases, The Second Xiangya Hospital of Central South University, Changsha, 410011 Hunan China; 2 Department of Metabolism and Endocrinology, The Affiliated Zhuzhou Hospital Xiangya Medical College CSU, Changsha, China

**Keywords:** Vitamin D, Triglyceride, Total cholesterol, LDL, HDL, Obesity

## Abstract

**Objective:**

The association between vitamin D deficiency and lipid profiles in adults with overweight or obesity remains unclear and inconsistent. The aim of our study was to determine the relationship between lipid profiles and vitamin D deficiency in the overweight and obese adults.

**Methods:**

Four databases, including PubMed, the Web of Science, EMBASE and the Cochrane Library, were used to identify all studies on vitamin D status and lipid levels, including the serum levels of triglycerides (TGs), total cholesterol (TC), low-density lipoprotein cholesterol (LDL), and high-density lipoprotein cholesterol (HDL). The Weighted mean difference (WMD) with 95% confidence intervals (CIs) using random-effects models was used to assess the association between the lipid profile and vitamin D deficiency.

**Results:**

Twenty-one articles that included a total of 7952 adults with overweight or obesity (BMI ≥ 25 kg/m2) were included. The overall results revealed that compared with the controls, individuals with vitamin D deficiency showed higher levels of TG (WMD = 15.01; 95%CI, 2.51–27.52) and TC (WMD = 8.61; 95%CI, 1.31–15.92). Moreover, vitamin D deficiency was related to an increased level of LDL (WMD = 6.12; 95%CI, 0.02–12.23). HDL level was inversely associated with the vitamin D deficiency status (WMD = -2.57; 95%CI, -4.26, -0.88).

**Conclusions:**

Among the adults with overweight or obesity, the vitamin D deficient group displayed impaired lipid profiles, including increased TG, TC and LDL levels and reduced HDL level.

**Supplementary Information:**

The online version contains supplementary material available at 10.1186/s12889-023-16447-4.

## Introduction

The rapidly increasing burden of obesity and its associated complications have become worldwide health issues [1]. There were over 650 million adults, approximately 13% of the worldwide adult population, were affected by obesity in 2016 [2]. The reciprocal relationship between micronutrient status and obesity and the increasing number of associated complications has been investigated in recent decades [3]. Paradoxically, individuals with obesity present micronutrient deficiency, including vitamin D deficiency, even though overnutrition and excessive energy absorption contribute to obesity [4]. Vitamin D, a liposoluble hormone, is mainly produced in response to ultraviolet light exposure and plays a significant role in various organs, including skeletal and nonskeletal tissues [5]. Vitamin D deficiency could potentially lead to insufficient insulin levels by disturbing insulin synthesis and secretion and accelerate the development of type 2 diabetes, obesity and metabolic syndrome [6]. Moreover, the prevalence of vitamin D deficiency would significantly increase in the overweight and obesity group compared with normal weight people, which are both worldwide health issues [7]. Vitamin D supplementation was also demonstrated to have a close relationship with weight gain and adiposity [8–10]. One of the underlying mechanisms of the association between the fat mass was proposed of which vitamin D may regulate the adipogenesis to effect body fat [8–10].

Vitamin D deficiency is known to be related with increasing obesity and body fat [11], little is known about the role of vitamin D deficiency on the extent and severity of obesity. Consistently, vitamin D deficiency was demonstrated to be related with atherosclerotic cardiovascular disease (CVD) and its dyslipidemia profile including levels of total cholesterol (TC), triglycerides (TGs), and low-density lipoprotein cholesterol (LDL-C) and high-density lipoprotein cholesterol (HDL-C) [12, 13], which have been one of the leading causes of death worldwide [14]. A growing number of studies have found that individuals with vitamin D deficiency tend to have poor lipid profiles [15–18]. In children and adolescents, higher vitamin D status was associated with an improved lipid profile in a recent meta-analysis [19]. Recently, a series of studies have argued that there may existed a relationship between vitamin D concentration and lipid profile in overweight and obese adults [20–22], while the results remain controversial and inconsistent. Moreover, to the best of our knowledge, no meta-analysis has been conducted for the association between lipid profiles and vitamin D deficiency in adults with obesity [23, 24]. The purpose of our study was to determine the role of vitamin D deficiency in the lipid profiles of adults with obesity by meta-analysis.

## Methods

This meta-analysis was performed according to the Preferred Reporting Items for Systematic Reviews and Meta-Analyses (PRISMA) statement guidelines, as previously described. The method descriptions were performed as we described previously [25].

### Article search strategy

We searched for eligible articles from January 25, 2022, to March 1, 2022. The PubMed (2013–2022, 1 March), Cochrane Library (1960–2022, 1 March), EMBASE (1960–2022, 1 March) and Web of Science (1950–2022, 1 March) databases were searched in this study. Searches for all published articles related to both vitamin D and lipid profiles were performed. The search strategy was as follows: (“Vitamin D” OR “Cholecalciferol” OR “Hydroxycholecalciferols:” OR “Ergocalciferols” OR “25-Hydroxyvitamin D” OR “Dihydrotachysterol”) AND (“Abdominal obesity” OR “Overweight” OR “Trunk obesity” OR “Android obesity” OR “Obesity” OR “Visceral Obesity” OR “Central obesity” OR “Central adiposity” OR “Central fat” OR “Anthropometric” OR “body mass index” or “BMI” OR “Waist circumference” OR “WC”). Additional papers were identified by performing manual searches of the references of relevant articles and tracking citations to obtain more relevant studies. All articles published by March 1, 2022, with no language restrictions were included.

### Selection criteria

Two reviewers (YY and XH) independently reviewed all eligible studies and selected those suitable for inclusion. Disagreements were settled by reaching a consensus or with the help of a third reviewer (JZ). Studies included in this meta-analysis if they met the following criteria: (1) had observational design (cohort, cross-sectional, or case–control studies); (2) were conducted on an adult population (18 years); (3) considered abdominal obesity or central obesity (waist circumference higher than 94 cm(male) or 80 cm(female)) or BMI greater or equal to 25 kg/m2 (BMI categories); (4) the control group had normal vitamin D levels; (5) a vitamin D deficiency group was required in the included articles; (6) importantly, the outcomes of the study had to refer to the lipid profiles of the different groups according to the status of vitamin D. Articles were excluded if they met the following criteria: (1) articles lacking information or data necessary for the purpose of this meta-analysis and (2) articles published as letters, reviews, editorials, or conference abstracts.

### Data extraction

All relevant articles were imported into EndNote X9 software and reviewed independently by two authors (YY and XH). Discrepancies between the authors were resolved with the help of a third reviewer (JZ). The following information was extracted from the selected studies by the two independent investigators: author, publication year, region, study design, mean or median age, sample size, and Newcastle–Ottawa Scale (NOS) scores. All extracted data were then imported into Excel.

### Quality assessment of studies

The quality of the included studies was assessed with the NOS [26]. We assessed the quality of all relevant studies based on the type of study, sample size, participant selection, representativeness of the sample, adequacy of follow-up, comparability (exposed-unexposed or case–control), and method of ascertaining the cases and controls. The possible range of NOS scores is from 0 to 9; studies that scored 4–6 represent a modest risk of bias, and those that scored < 3 indicate the highest risk of bias. A study with a score higher than 6 was defined as high-quality.

### Statistical analysis

All analyses were performed using Stata (Version 13.0). The association between vitamin D deficiency and lipid levels in individuals with obesity was expressed as the pooled weighted mean difference (WMD) and 95% CI. A random-effects model was used for all results of our meta-analysis. I^2^ statistics were used to assess the degree of heterogeneity as follows: 25%, 50%, and 75% represented low, moderate, and high degrees of heterogeneity, respectively. Subgroup analyses were performed using the following variables to analyze the heterogeneity: Gender (Male/Female or Female), Age (< 40 years or > 40 years), BMI (< 35 kg/m^2^ or > 35 kg/m^2^), Publication year (Before 2015 or After 2016) and Definition of vitamin D deficiency (< 22-49 nmol/l or < 50-75 nmol/l). Meta-regression were performed to analyze the source of heterogeneity, with a *p* value < 0.05 suggesting significance. The sensitivity analysis would be used the metanif package and leave1out function. Additionally, funnel plots showed no detected potential publication bias.

## Results

### Search results and study characteristics

**Fig. 1:**
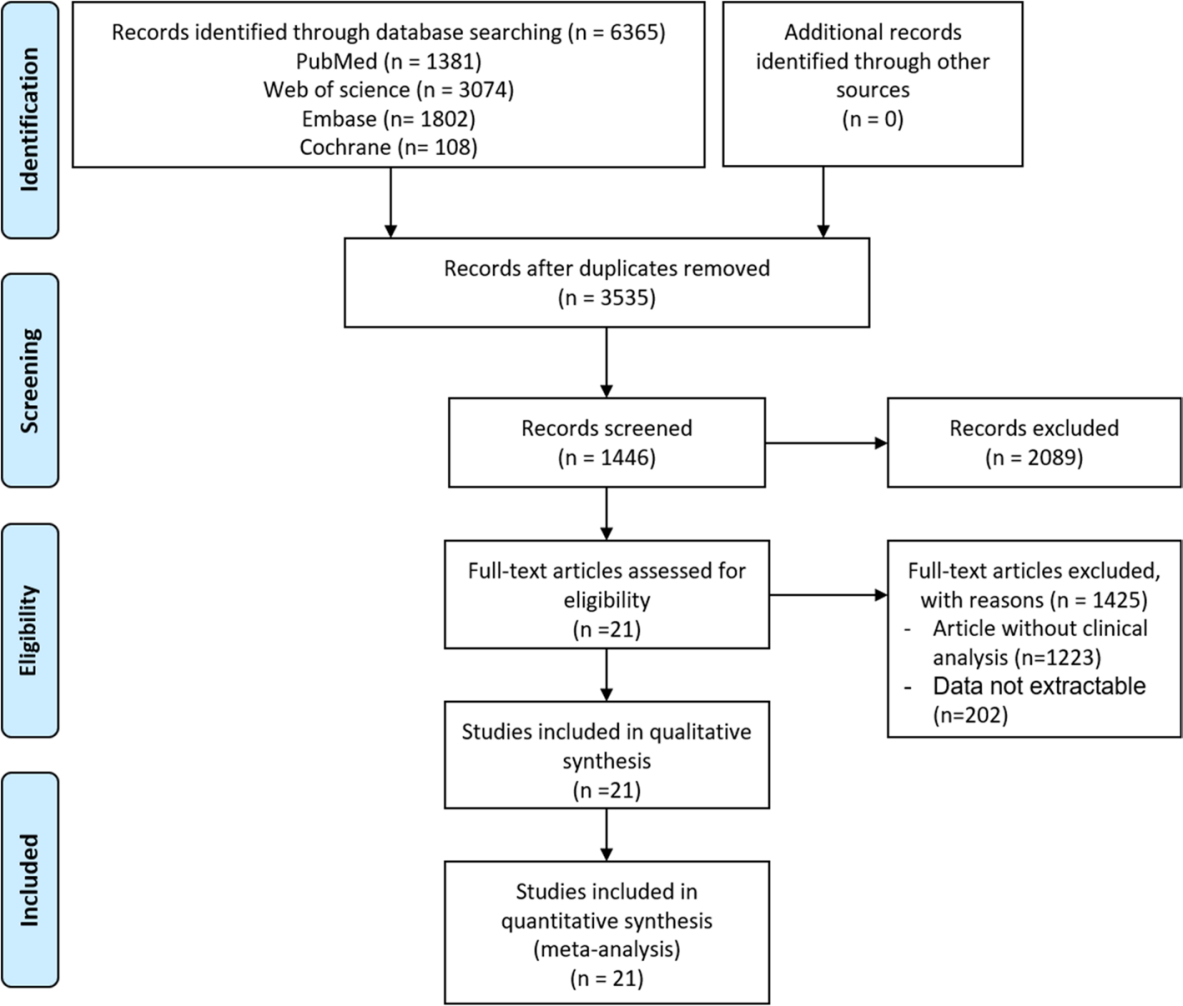
Flow diagram of the study selection process

The study selection process of this meta-analysis is displayed in Fig. 1. A total of 6365 studies were identified. After 2830 duplicates were eliminated, 3535 studies remained. We further excluded 2089 ineligible studies by screening the titles and abstracts. Of the remaining 1446 articles, 1425 studies were further removed for the following reasons: (1) articles without enough clinical information (*n* = 1223); and (2) the original data regarding lipid profiles were not extractable (*n* = 202). Finally, 21 eligible articles related to vitamin D and lipid levels were included in this meta-analysis (Fig. 1). The detailed characteristics of the 21 eligible studies [20–22, 27–44] are shown in Table 1. Among the 21 studies included in this analysis, 3 studies were performed in China; 3, in the USA; 4, in Italy; 1, in Turkey; 1, in Spain; 3, in Brazil; 1, in Australia; 1, in Poland; 1, in Ajman; 2, in Iran; and 1 in United Arab Emirates (Table 1). Given that various medication could be the significant confounding factor for vitamin D level, especially the lipid-lowering drugs, we examined the drugs using in the included studies. However, the most included researches have not controlled for the use of such agents, only 7 studies have mentioned that the usage of such drugs. In details, 5 studies demonstrated that the hypolipidemic drugs were controlled, and 1 study has 41.2% participants were receiving anti-diabetes drugs and statins, and 1 research excluded usage of antihypertensive drugs.

**Table 1:** Description of eligible studies reporting the association between vitamin D deficiency and lipid profiles

No	Author,year	Gender	Specific population	Methods of vitamin D measurement	Region	Study type	Age (years)	BMI (kg/m2)	Sample Size	NOS
1	**Carretero 2007** [27]	**male/female (13/60)**	**morbidly obese patients**	ELISA	Spain	cohort	39.0 ± 12.7	48.6 ± 5.8	73	7
2	**Yildizhan 2009** [28]	**women**	**obese women with polycystic ovary syndrome**	high-performance liquid chromatography (HPLC)-based Chromsystems diagnostic kit	Turkey	prospective study	25.51 ± 3.91	32.84 ± 5.43	57	7
3	**Muscogiuri 2010** [29]	**male/female (11/21)**	**obese subjects**	chemiluminescence immunoassay radioimmunoassay	Italy	cohort	41.4 ± 12.4	30.1 ± 5.4	39	6
4	**Bellia 2013** [30]	**male/female (58/89)**	**patients with severe obesity**	ND	Italy	cohort	37 ± 10	45.1 ± 2.2	147	7
5	**Esteghamati 2014** [31]	**male/female (142/124)**	**metabolically healthy and unhealthy obese adults**	Radioimmunoassay kits	Iran	cross-sectional	47.2 ± 12.3	29.6 (29.4–29.8)	4391	7
6	**Boonchaya-anant 2014** [32]	**male/female (41/150)**	**extremely obese individuals**	a TSQ Quantum Ultra triple mass-spectrometer	USA	a retrospective study	41.5 ± 11.2	40.9 ± 9.1	63	8
7	**Kozakowski 2014 [**34**]**	**women**	**women with polycystic ovary syndrome presenting abdominal and gynoidal type of obesity**	a chemiluminescent immunoassay	Poland	cohort	30.2 ± 8.8	36.6 ± 4.8	26	6
8	**Amena Sadiya 2014** [33]	**male/female (79/230)**	**persons with obesity and type 2 Diabetes**	an immunochemiluminescence method	Ajman	cross-sectional	48.7 ± 7.8	36.9 ± 6.0	309	7
9	**Bellan 2014** [35]	**male/female (221/303)**	**patients with severe obesity**	ND	Italy	cohort	52.0 (40.0–62.0)	46.6 (43.1–50.9)	524	6
10	**Bril 2015** [36]	**male/female (204/35)**	**patients with non-alcoholic steatohepatitis**	chemiluminescence immunoassay	USA	cohort	59 ± 1	34.3 ± 0.4	239	9
11	**Lu 2015** [37]	**women**	**Chinese Postmenopausal Women with visceral obesity**	electrochemiluminescence immunoassay	China	cross-sectional	56.96 ± 4.27	25.28 ± 2.68	226	6
12	**Ter horst 2016** [38]	**women**	**obese women**	iso-tope dilution liquid chromatography–tandem mass spectrometry	USA	cohort	44 ± 9	46 ± 7	37	6
13	**Mousa 2017** [39]	**male/female (66/45)**	**overweight/obese but otherwise healthy cohort**	the direct competitive chemiluminescent immunoassay method	Australia	cohort	28 (23–37)	29.6 (27–33)	111	7
14	**Piantanida 2017** [40]	**male/female (44/152)**	**people with visceral obesity**	ND	Italy	cohort	46 ± 14	36.3 ± 4	196	6
15	**Ong 2018** [41]	**male/female (54/57)**	**Overweight and Obese Singaporeans Seeking Weight** **Management Including Bariatric Surgery**	chemiluminescent immunoassay	China	cross-sectional	40 ± 10	40.1 ± 8.2	111	7
16	**Curvello-Silva 2020 [**42**]**	**male/female (75/224)**	**obese patients**	chemiluminescence immunoassay	Brazil	cross-sectional	36.0 ± 9	40.8 ± 5.1	299	6
17	**Setayesh 2021** [43]	**women**	**overweight and obese women**	ELISA	Iran	cross-sectional	35.61 ± 8.17	31.14 ± 4.14	236	7
18	**Minna F. Schleu 2021** [22]	**women**	**obese Brazilian Women**	ELISA	Brazil	cross-sectional	44 (33–53.5)	37.755 (33.74–41.16)	93	6
19	**Lara A da C. Dominoni 2022** [21]	**male/female (20/32)**	**adults with obesity**	chemiluminescent microparticle immunoassay	Brazil	cross-sectional	37.50 ± 6.88	33.60 ± 2.89	52	6
20	**Salah Gariballa 2022** [20]	**male/female (142/56)**	**obese subject**	Chemiluminescence immunoassay	United Arab Emirates	cohort	41 ± 12	≥ 30	277	7
21	**Tong Gong 2022** [44]	**male/female (293/153)**	**overweight/obese patients with type 2 diabetes**	ND	China	cross-sectional	50.76 ± 13.31	25.52 ± 3.78	446	6

Data are presented as mean (standard deviation, S.D.)

*NOS* Newcastle–Ottawa Scale, *ND* Not Determined

### Quality assessment

The NOS mainly consists of the following three aspects: sample selection, comparability of cases and controls, and exposure. All included studies had NOS scores higher than 6, indicating the high quality of our studies. The details of the risk of bias are described in Table 2.

**Table 2:** Details of Newcastle–Ottawa Scale (NOS)

NOS items / Study ID	Is the case definition adequate?	Representativeness of the cases	Selection of controls	Definition of controls	Compatibility	Ascertainment of Exposure	Same method of ascertainment for cases and control	Non-response Rate	Total Score
Carretero 2007 [27]	*	*	*	*	**		*	*	8
Yildizhan 2009 [28]		*		*	*	*	*	*	6
Muscogiuri 2010 [29]	*	*	*	*	**			*	7
Bellia 2013 [30]	*	*	*	*	**	*	*	*	9
Esteghamati 2014 [31]	*	*	*	*		*	*	*	7
Boonchaya-anant 2014 [32]	*	*	*	*	**			*	7
Kozakowski 2014 [34]	*	*	*	*	**	*	*		8
Amena Sadiya 2014 [33]	*	*			**	*	*	*	7
Bellan 2014 [35]	*	*	*	*				*	6
Bril 2015 [36]	*	*	*	*	**	*	*	*	9
Lu 2015 [37]	*			*	**	*	*	*	7
Ter horst 2016 [38]		*	*	*		*	*	*	6
Mousa 2017 [39]	*	*	*	*	**				6
Piantanida 2017 [40]	*	*	*	*		*	*	*	7
Ong 2018 [41]	*	*		*	**	*	*	*	8
Curvello-Silva 2020 [42]	*	*	*		**			*	6
Setayesh 2021 [43]	*	*	*			*	*	*	6
Minna F. Schleu 2021 [22]	*	*	*	*	**	*			7
Lara A da C. Dominoni 2022 [21]			*	*	*	*	*	*	6
Salah Gariballa 2022 [20]	*	*	*	*	*	*		*	7
Tong Gong 2022 [44]	*	*		*	*	*	*	*	7

One asterisk presents one score, higher scores indicating higher quality of study. A study can be awarded a maximum of one asterisk for each numbered item excepts Comparability, a maximum of two asterisk can be given for Comparability

### Vitamin D and TG

**Fig. 2:**
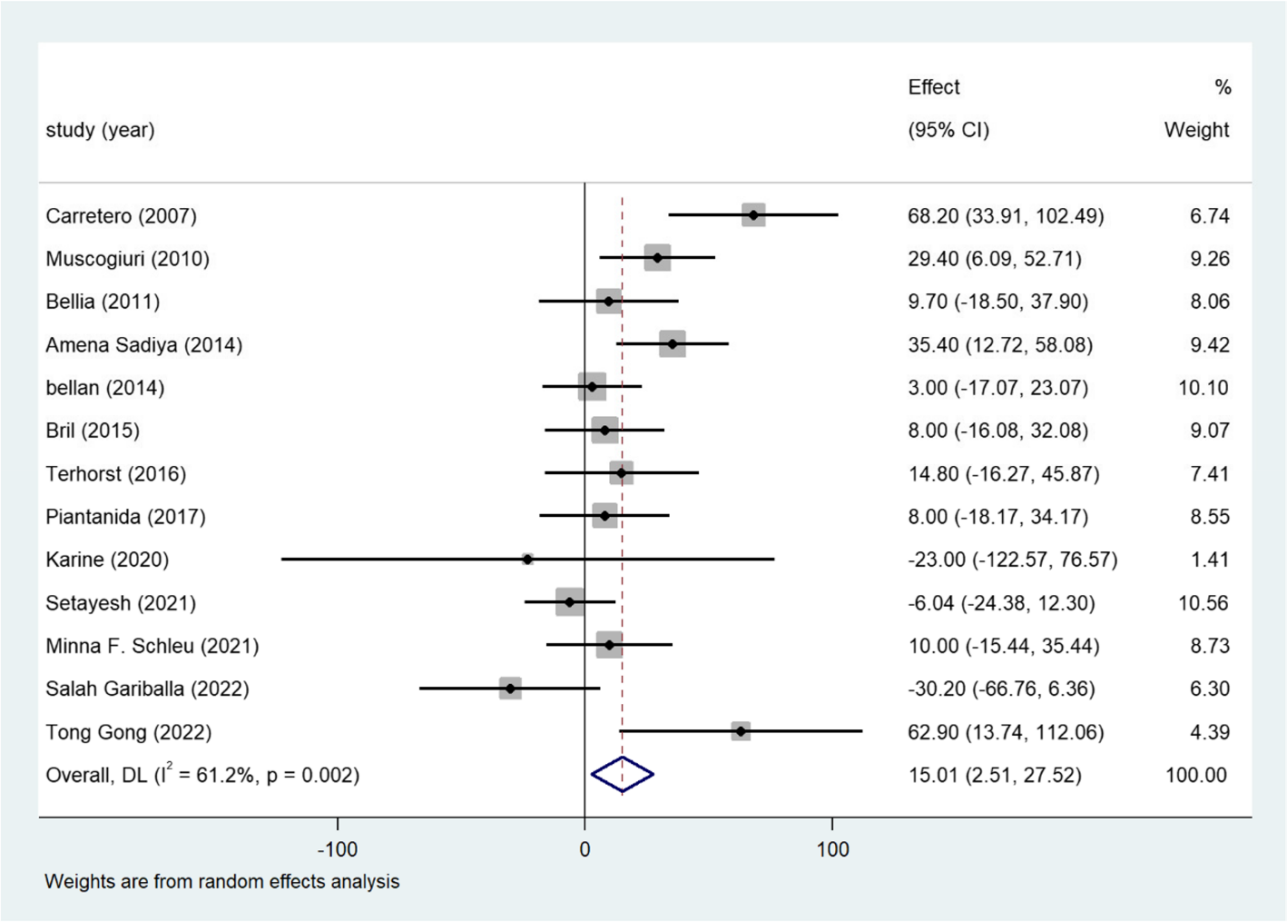
Forest plots of WMD for the association between vitamin D deficiency and TG

The 21 included studies, 13 studies reported TG levels in patients with vitamin D-deficient (Fig. 2). The overall results revealed that compared with that in the normal vitamin D group, the TG concentration in the vitamin D deficiency group was significantly higher (WMD = 15.01; 95%CI, 2.51–27.52). Strong heterogeneity existed in this result (I^2^ statistic = 61.2%, *p* = 0.002). Subgroup analysis based on gender, age, BMI, publication year, definition of vitamin D deficiency were conducted, but the heterogeneity was still high in the subgroups, meta-regression showed no association between the variances and mean difference of serum TG levels (Table S3). Funnel plots (Fig. S1A) displayed a symmetrical distribution. A sensitivity analysis was further conducted and showed that our result was stable (Fig. S1B).

### Vitamin D and TC

**Fig. 3:**
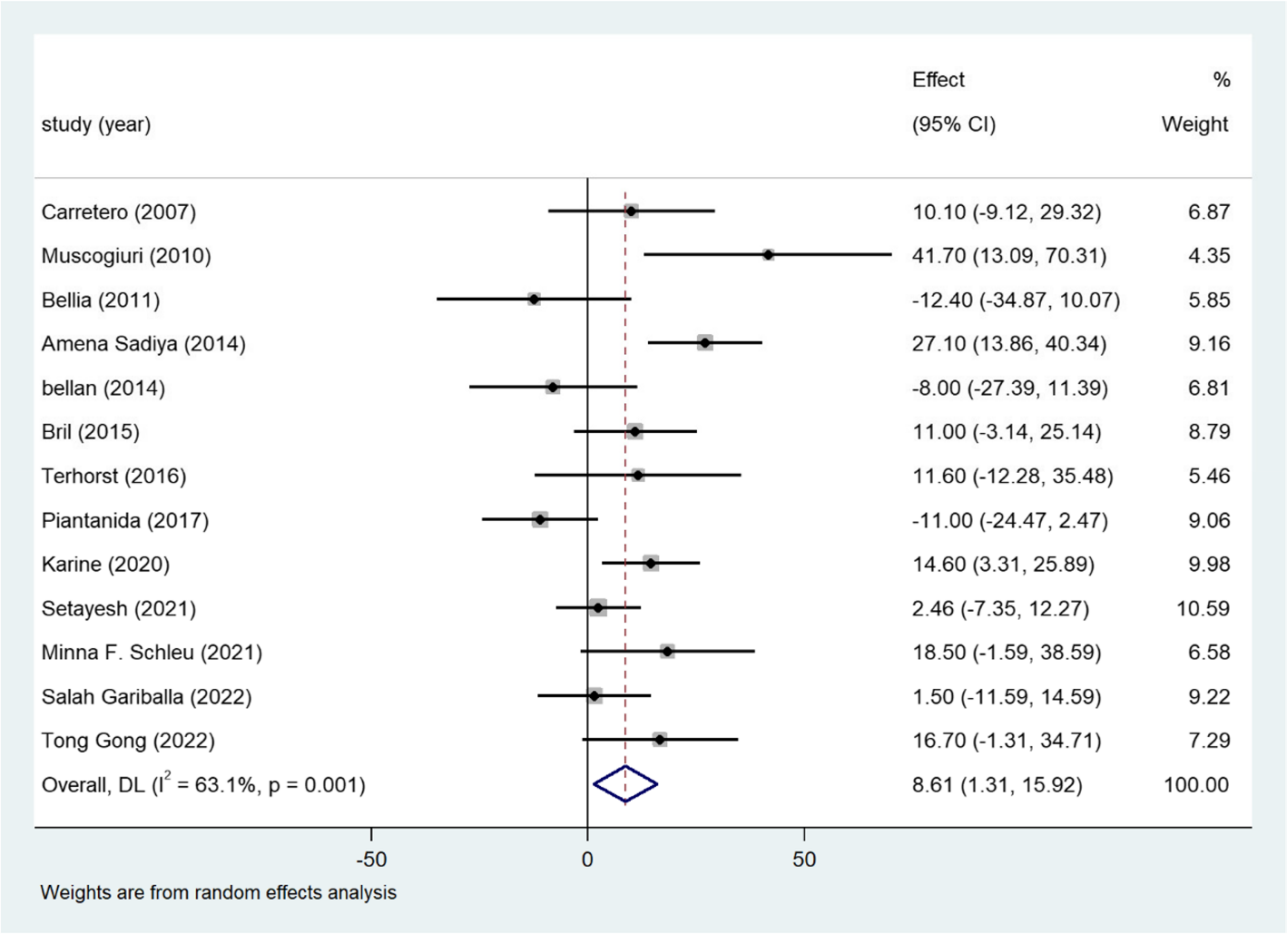
Forest plots of WMD for the association between vitamin D deficiency and TC

As another significant indicator of the lipid profile, the level of TC was examined in this part. The primary results of 13 studies revealed a positive correlation between vitamin D deficiency and high TC levels (WMD = 8.61; 95%CI, 1.31–15.92) (Fig. 3). High heterogeneity existed among the included studies (I^2^ statistic = 63.1%, *p* = 0.001). Subgroup analysis based on gender, age, BMI, publication year, definition of vitamin D deficiency were conducted, but the heterogeneity was still high in the subgroups, meta-regression showed no association between the variances and mean difference of serum TC levels (Table S4). The funnel plot displayed a symmetrical distribution (Fig. S2A). A normal result of the sensitivity analysis was also obtained for this outcome (Fig. S2B).

### Vitamin D and LDL

**Fig. 4:**
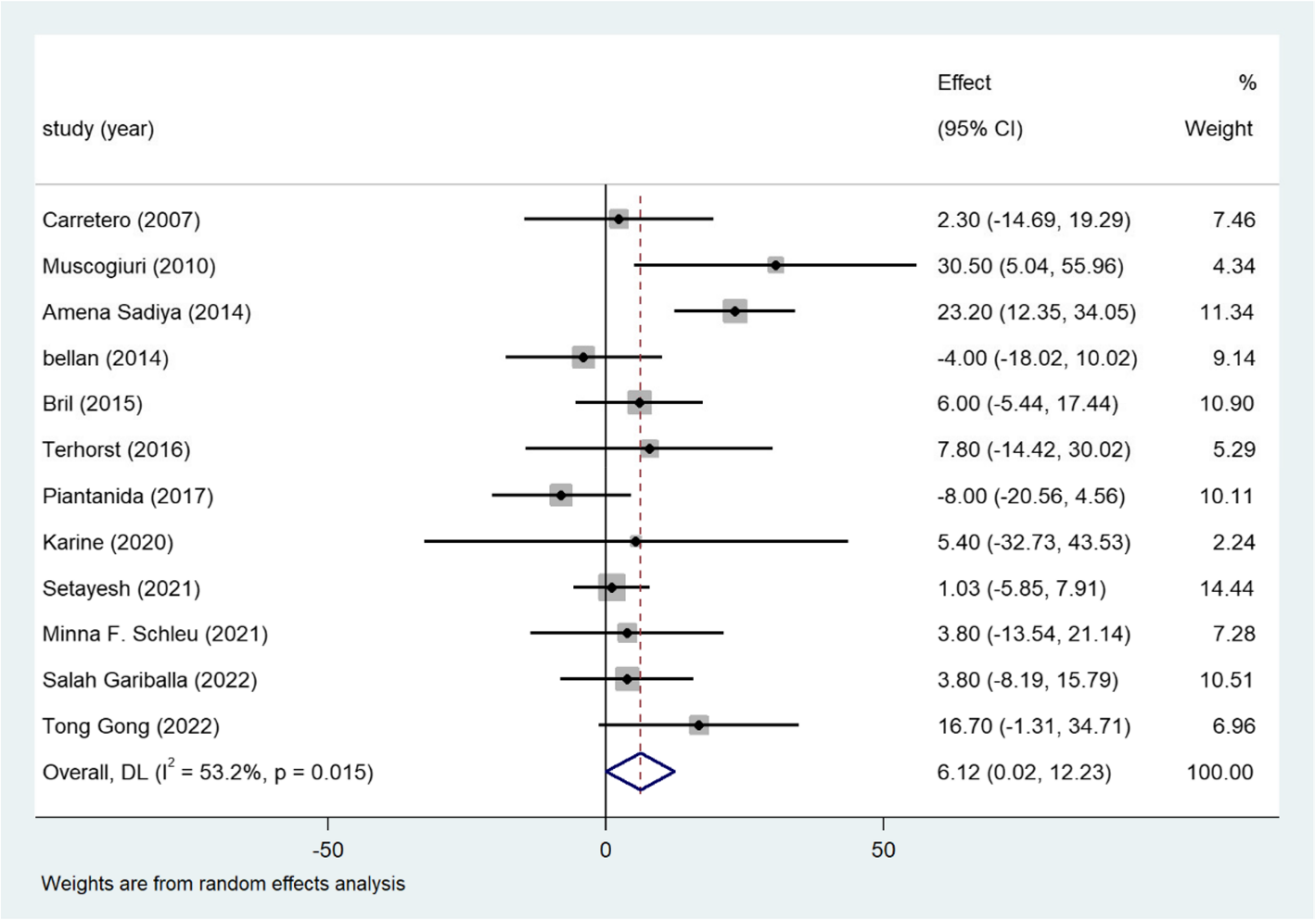
Forest plots of WMD for the association between vitamin D deficiency and LDL

Twelve studies reported LDL levels under vitamin deficiency conditions. There was no significant difference between the control and deficiency groups (WMD = 6.12; 95%CI, 0.02–12.23) (Fig. 4). Mild heterogeneity existed in this result (I^2^ statistic = 38.3%, *p* = 0.078), subgroup analysis based on gender, age, BMI, publication year, definition of vitamin D deficiency were conducted, but the heterogeneity was still high in the subgroups, meta-regression showed no association between the variances and mean difference of serum LDL levels (Table S5). However, the funnel plot showed a mild asymmetric distribution (Fig. S3A). The sensitivity analysis conducting on this part is still stable (Fig. S3B).

### Vitamin D and HDL

**Fig. 5:**
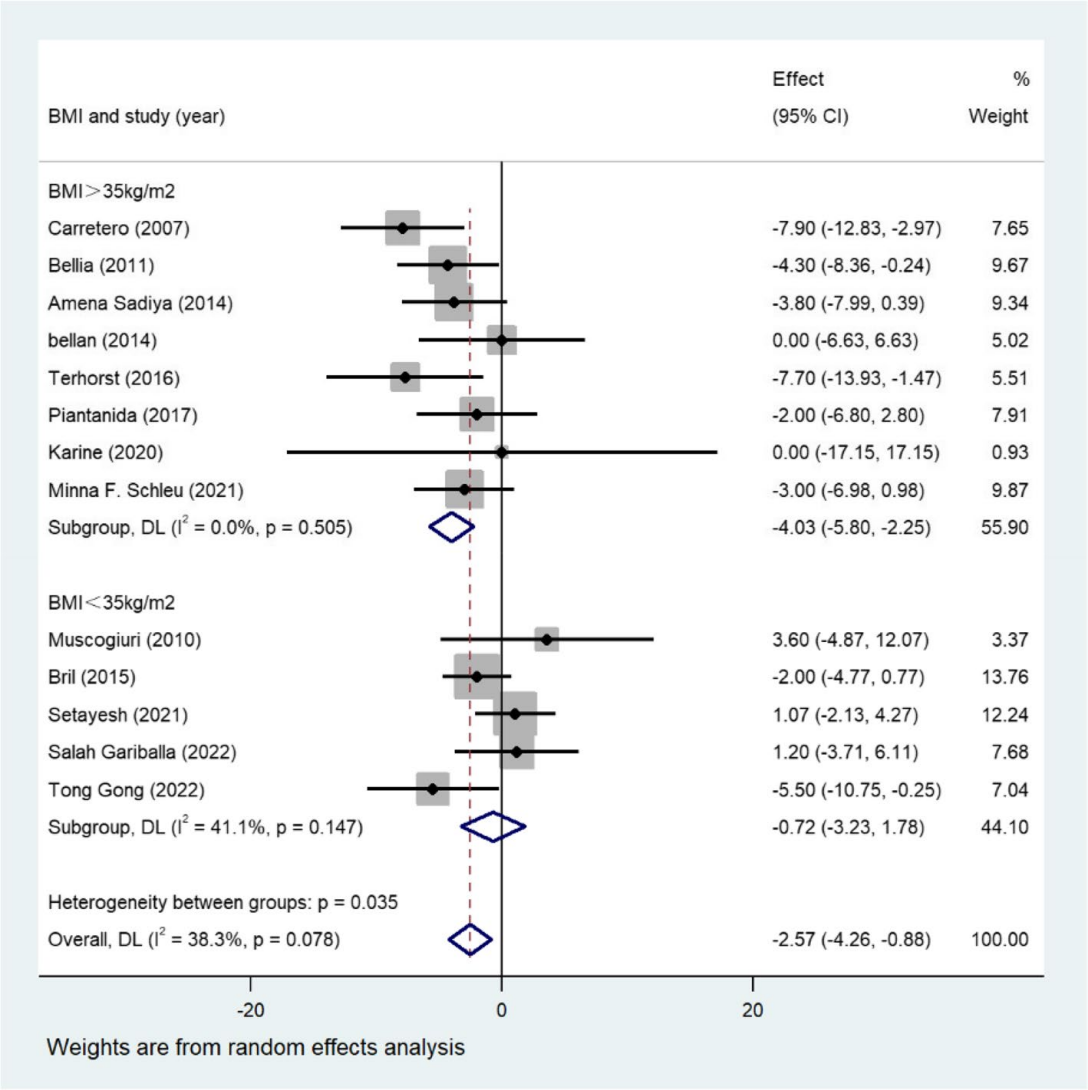
Forest plots of WMD for the association between vitamin D deficiency and HDL

A total of 13 eligible studies assessed HDL levels in adults with vitamin D-deficient. The primary results demonstrated that HDL level was lower in vitamin D-deficient groups (WMD = -2.57; 95%CI, -4.26, -0.88) (Fig. 5). Mild heterogeneity existed in this result (I2 statistic = 38.3%, *p* = 0.078). Subgroup analysis based on BMI showed the significance between HDL concentration and vitamin D deficiency in population of BMI > 35 kg/m^2^ (WMD = -4.03; 95%CI, -5.80, -2.25), but the not the subgroup of BMI < 35 kg/m^2^ (WMD = -0.72; 95%CI, -3.23, 1.78), and the meta-regression showed significance between subgroups (Table S4). Funnel plots also suggested that no publication bias existed (Fig. S4A). Sensitivity analysis also revealed the stability of our results (Fig. S4B).

### Correlation coefficients between serum 25(OH)D levels and lipid profiles

There are twelve studies indicating the correlation coefficients with or without *p* values between vitamin D and lipid levels (Table 3). Among the included 12 studies, we found that there were different results in various populations. Interestingly, two included studies [33, 44] conducted in the persons with obesity and type 2 diabetes has the correlation coefficients which reached the statistical significance. Additionally, all the three studies performed in the women with obesity [28, 34, 37] demonstrated that the significant inverse association between serum 25(OH)D and triglycerides was existed, while other lipid profiles including TC, LDL and HDL showed no statistical significance. Apart from the above two populations, the residual seven studies [20, 21, 30, 32, 39, 41, 42] indicating that serum 25(OH)D showed no clear correlation with any lipid profiles in the overweight/obesity population without diabetes complications.

**Table 3:** Correlation coefficients between serum 25(OH)D levels and lipid profiles for included studies

No	Study	Population	n	correlation coefficient	Triglycerides		Total cholesterol		LDL		HDL	
					r	*p* value	r	*p* value	r	*p* value	r	*p* value
1	Yildizhan 2009 [28]	obese and women with PCOS	57	Pearson’s correlation	-0.990	**0.01**	-0.207	NA	NA	NA	NA	NA
2	Bellia 2013 [30]	severe obese subjects	147	a multivariate regression analysis (95% CI)	0.0023 (-0010 to 0.0099)	0.543	-0.0013 (-0.0044 to 0.0014)	0.473	NA	NA	0.0005 (-0.001 to 0.0015)	0.642
3	Boonchaya-anant 2014 [32]	Extremely Obese Individuals	191	Pearson’s correlation	-0.004	0.959	0.043	0.579	0.036	0.644	0.085	0.271
4	Kozakowski 2014 [34]	women with polycystic ovary syndrome presenting abdominal and gynoidal type of obesity	26	Pearson’s correlation	abdominal: -0.97; gynoidal:-0.19	**abdominal: *p* < 0.01;** gynoidal:*p* > 0.05	abdominal: -0.4; gynoidal:-0.21	*p* > 0.05	abdominal: -0.42; gynoidal:-0.42	*p* > 0.05	abdominal: 0.65; gynoidal:0.33	*p* > 0.05
5	Amena Sadiya 2014 [33]	persons with obesity and type 2 Diabetes	309	Pearson’s correlation	-0.15	**0.01**	-0.16	***p* < 0.01**	-0.16	*p* < 0.01	0.12	***p* = 0.03**
6	Lu 2015 [37]	Chinese Postmenopausal Women with visceral obesity	226	partial correlation analysis	-0.127	**0.007**	-0.082	0.082	0.02	0.675	0.02	0.675
7	Mousa 2017 [39]	overweight/obese but otherwise healthy cohort	111	Pearson’s correlation	-0.07	0.5	-0.09	0.3	-0.13	0.2	0.05	0.6
8	Ong 2018 [41]	Overweight and Obese Singaporeans Seeking Weight Management Including Bariatric Surgery	111	Pearson’s correlation	-0.14	*p* > 0.05	0.06	*p* > 0.05	0.05	*p* > 0.05	0.177	*p* > 0.05
9	Curvello-Silva 2020 [42]	obese patients	299	Pearson’s correlation	-0.022	0.778	-0.157	0.047	-0.164	0.038	-0.023	0.769
10	Lara A da C. Dominoni 2022 [21]	adults with obesity	52	A regression coefficient (ß, 95% CI)	–0.794 CI:–3.19 to 1.60	0.508	–0.477 CI: –1.92 to 0.97	0.511	–0.251 CI:–1.53 to 1.03	0.696	–0.144 CI:–0.76 to 0.48	0.644
11	Salah Gariballa 2022 [20]	obese subject	277	Regression coefficient (95% CI)	0.001 (-0.011 to 0.014)	0.864	NA	NA	-0.006 (-0.015 to 0.004)	0.241	0.001 (-0.003 to 0.005)	0.564
12	Tong Gong 2022 [44]	overweight/obese patients with type 2 diabetes	446	Pearson’s correlation	-0.322	***p* < 0.001**	-0.129	0.029	**0.01**	0.871	0.165	**0.005**

Data are given as Pearson’s correlation (r = correlation coefficient)

*ND* Not Determined, *LDL* low-density lipoprotein cholesterol, *HDL* high-density lipoprotein cholesterol

## Discussion

Recent clinical studies have revealed a significant association between obesity and vitamin D deficiency [20, 30, 33, 45]. Regarding both the prevalence of obesity and vitamin D deficiency, which is alarmingly increasing worldwide, there is a strong interest in studying all the important aspects and the underlying pathophysiological mechanisms of this association [45–47].

### Association between vitamin D and lipid profile in adults with obesity

Twenty-one eligible studies with 7952 adults with obesity were included in our meta-analysis. Vitamin D deficiency was related to higher TG concentrations in participants with overweight or obesity (Fig. 2). Approximately 13 studies reported the TC level, which revealed a positive association between TC and vitamin D deficiency in adults with obesity (Fig. 3). Consistent with these findings, another 12 studies confirmed the inverse relationship between LDL and low vitamin D levels (Fig. 4). Additionally, a significant inverse association between HDL and vitamin D deficiency has been found thus far (Fig. 5). Additionally, results of correlation coefficients between serum 25(OH)D levels and lipid profiles (Table 3) demonstrated that populations of the obesity combined with T2DM or the obesity in women may be the possible risk factor for dyslipidemia rather than the overweight/obesity population without diabetes complications, indicating that the relationship between serum 25(OH)D levels and lipid profiles may be various in obese population with different characteristics. However, considering the limited sample size and included clinical studies of the correlation coefficients, more studies are urgent to improve these results.

There is a significant impact of lots of medication on vitamin D status including metformin, statins, calcium channel blockers, digoxin, lipase inhibitors, bile acid sequestrants, loop diuretics, angiotensin-converting enzyme inhibitors, thiazide diuretics, antagonists of vitamin K, potassium-sparing diuretics, benzodiazepines, antidepressants, proton pump inhibitors, et al. [48]. Only 7 studies have excluded the possible medication including lipid-lowing drugs and antihypertensive drugs, while the other included studies have not mentioned the different drugs the patients taken, which make our results remain defects and flaws. Apart from the medication use, the level of vitamin D was also affected by the dietary intake, vitamin D supplementation, periodic religious fasting and sunlight exposure [49, 50], while most recent researches have not provided detailed information about these, which making the results still remain bias factors. Given that the data on information including medication use, dietary intake, seasoning and sunlight exposure of most included studies is heterogeneous, more well-designed clinical researches are needed to exclude the possible effect of these factors on the level of vitamin D. Additionally, most of the data on sex of included participant are the mixture of male and female, while it is known that HDL-C levels are strongly influenced by gender, more well-designed articles are requested to improve the relationship between vitamin-D deficiency and lipid profile in the overweight or obese adults.

### Mechanism underlying the relationship between vitamin D and the lipid profile

The mechanism underlying the relationship between vitamin D and the lipid profile remains unclear, but there are several explanations. First, vitamin D deficiency leads to calcium spillage into fat cells and thus increases lipogenesis by promoting hyperparathyroidism [51]. Moreover, the elevated level of calcium ions in fat cells contributes to higher serum fatty acid synthase, which is responsible for inhibiting lipolysis and lipid deposition [52]. In addition, higher vitamin D status was demonstrated to improve the level of leptin, subsequently improving lipolysis and reducing lipogenesis [46]. Additionally, vitamin D deficiency contributes to impaired islet function and insulin resistance, which also leads to an impaired lipid profile [53]. Recently, vitamin D deficiency was shown to regulate lipid metabolism by inhibiting significant regulators of lipogenesis, including sterol regulatory element-binding protein (SREBP) and SREBP cleavage-activating protein (SCAP) [54]. The function of vitamin D upregulating the lipoprotein lipase expression may also partly explain the relationship of with lipid profile and vitamin D [55]. Adiposity was known to impair lipolysis of TG-rich lipoproteins by reducing the lipoprotein lipase expression in adipose tissue [56], hypertriglyceridemia correlate with the formation of small dense of LDL [57] and the dissociation of cholesterol esters from HDL [58], ultimately lead to higher levels of LDL and lower levels of HDL. Vitamin D deficiency in overweight or obese adults may further aggravate the above process of lipolysis by inhibiting the lipoprotein lipase expression. Another possible mechanism may include the relationship between hyperparathyroidism and dyslipidemia. Hyperparathyroidism was also an important confounding factors for serum vitamin D deficiency [59]. And the hyperparathyroidism was related with the dyslipidemia, while the concrete mechanism still remain unclear [60]. The relationship between vitamin D deficiency with dyslipidemia may be partly affected by hyperparathyroidism.

## Limitations of the study

There are several limitations of our study. First, the heterogeneity in the results cannot be ignored, although the subgroup analysis and the meta-regression have been conducted (Tables S3, S4, S5, S6). And our study was not registered online, which was also one of our flaws and limitations. Second, the results from this study are based only on observational studies, which have many confounders; therefore, the evidence generated from this study is not strong enough. More randomized clinical trials are still needed to confirm the results from this study. Thirdly, specific populations were included in 21 selected studies (e.g., postmenopausal/perimenopausal women, women with PCOS, patients on dialysis, patients with Type 2 diabetes mellitus, patients with non-alcoholic steatohepatitis, apparently healthy subjects, older adults, younger adults) (Table 1). Therefore, this was not homogeneous sample to allow such meta-analysis. Additionally, more prospective studies are required to clarify whether there is a causal relationship between vitamin D deficiency and lipid profile in the overweight or obese adults. It is also necessary to adjust statistical analyses for season and age, while the most included studies have missed this part, more well-designed clinical studies and updated meta-analysis are needed to improve these limitations.

## Conclusion

Based on observational clinical studies, our meta-analysis demonstrated that vitamin D deficiency was associated with higher TG, TC and LDL levels and lower HDL level in adults with obesity.

### Supplementary Information

**Additional file 1.** 

**Additional file 2.** 

**Additional file 3.**

## Data Availability

All data generated or analyzed during the present study are included in this published article.

## References

[1] Ford ND, Patel SA, Narayan KM (2017). Obesity in low- and middle-income countries: burden, drivers, and emerging challenges. Annu Rev Public Health.

[2] WHO. Obesity and overweight. Aug 2019; Available from: https://www.who.int/news-room/fact-sheets/detail/obesity-and-overweight.

[3] Tamer G (2012). Is vitamin D deficiency an independent risk factor for obesity and abdominal obesity in women?. Endokrynol Pol.

[4] Via M (2012). The malnutrition of obesity: micronutrient deficiencies that promote diabetes. ISRN Endocrinol.

[5] Webb AR, Holick MF (1988). The role of sunlight in the cutaneous production of vitamin D3. Annu Rev Nutr.

[6] Nam GE (2012). Estimate of a predictive cut-off value for serum 25-hydroxyvitamin D reflecting abdominal obesity in Korean adolescents. Nutr Res.

[7] de Oliveira LF (2020). Obesity and overweight decreases the effect of vitamin D supplementation in adults: systematic review and meta-analysis of randomized controlled trials. Rev Endocr Metab Disord.

[8] Pathak K (2014). Vitamin D supplementation and body weight status: a systematic review and meta-analysis of randomized controlled trials. Obes Rev.

[9] Mallard SR, Howe AS, Houghton LA (2016). Vitamin D status and weight loss: a systematic review and meta-analysis of randomized and nonrandomized controlled weight-loss trials. Am J Clin Nutr.

[10] Lotito A, et al. Serum parathyroid hormone responses to vitamin D supplementation in overweight/obese adults: a systematic review and meta-analysis of randomized clinical trials. Nutrients. 2017;9(3):241. 10.3390/nu9030241 PMC5372904 28272298

[11] Pereira-Santos M (2015). Obesity and vitamin D deficiency: a systematic review and meta-analysis. Obes Rev.

[12] Dobnig H (2008). Independent association of low serum 25-hydroxyvitamin d and 1,25-dihydroxyvitamin d levels with all-cause and cardiovascular mortality. Arch Intern Med.

[13] Martini LA, Wood RJ (2006). Vitamin D status and the metabolic syndrome. Nutr Rev.

[14] Murray CJ (2012). Disability-adjusted life years (DALYs) for 291 diseases and injuries in 21 regions, 1990–2010: a systematic analysis for the Global Burden of Disease Study 2010. Lancet.

[15] Ponda MP (2012). Vitamin D may not improve lipid levels: a serial clinical laboratory data study. Circulation.

[16] Vacek JL (2012). Vitamin D deficiency and supplementation and relation to cardiovascular health. Am J Cardiol.

[17] Jorde R (2010). High serum 25-hydroxyvitamin D concentrations are associated with a favorable serum lipid profile. Eur J Clin Nutr.

[18] Skaaby T (2012). Vitamin D status and changes in cardiovascular risk factors: a prospective study of a general population. Cardiology.

[19] Kelishadi R, Farajzadegan Z, Bahreynian M (2014). Association between vitamin D status and lipid profile in children and adolescents: a systematic review and meta-analysis. Int J Food Sci Nutr.

[20] Gariballa S (2022). Vitamin D [25(OH)D] metabolites and epimers in obese subject: Interaction and correlations with adverse metabolic health risk factors. J Steroid Biochem Mol Biol.

[21] Dominoni L (2022). Vitamin D is associated with body composition and fat intake, but not with cardiometabolic parameters in adults with obesity. Nutr Res.

[22] Schleu MF, et al. Lower levels of vitamin D are associated with an increase in insulin resistance in obese brazilian women. Nutrients. 2021;13(9):2979. 10.3390/nu13092979 PMC8471993 34578857

[23] Chacko SA (2011). Serum 25-hydroxyvitamin D concentrations in relation to cardiometabolic risk factors and metabolic syndrome in postmenopausal women. Am J Clin Nutr.

[24] O’Hartaigh B, et al. Association of 25-hydroxyvitamin D with type 2 diabetes among patients undergoing coronary angiography: cross-sectional findings from the LUdwigshafen Risk and Cardiovascular Health (LURIC) Study. Clin Endocrinol (Oxf). 2013;79(2):192–8. 10.1111/cen.12024 22924597

[25] Yang Y, Cai Z, Zhang J (2021). Insulin treatment may increase adverse outcomes in patients with COVID-19 and diabetes: a systematic review and meta-analysis. Front Endocrinol (Lausanne).

[26] Stang A (2010). Critical evaluation of the Newcastle-Ottawa scale for the assessment of the quality of nonrandomized studies in meta-analyses. Eur J Epidemiol.

[27] Botella-Carretero JI (2007). Vitamin D deficiency is associated with the metabolic syndrome in morbid obesity. Clin Nutr.

[28] Yildizhan R (2009). Serum 25-hydroxyvitamin D concentrations in obese and non-obese women with polycystic ovary syndrome. Arch Gynecol Obstet.

[29] Muscogiuri G (2010). 25-Hydroxyvitamin D concentration correlates with insulin-sensitivity and BMI in obesity. Obesity (Silver Spring).

[30] Bellia A (2013). Serum 25-hydroxyvitamin D levels are inversely associated with systemic inflammation in severe obese subjects. Intern Emerg Med.

[31] Esteghamati A (2014). Differences in vitamin D concentration between metabolically healthy and unhealthy obese adults: associations with inflammatory and cardiometabolic markers in 4391 subjects. Diabetes Metab.

[32] Boonchaya-anant P, Holick MF, Apovian CM (2014). Serum 25-hydroxyvitamin D levels and metabolic health status in extremely obese individuals. Obesity (Silver Spring).

[33] Sadiya A (2014). Vitamin D status and its relationship with metabolic markers in persons with obesity and type 2 diabetes in the UAE: a cross-sectional study. J Diabetes Res.

[34] Kozakowski J, Kapuścińska R, Zgliczyński W (2014). Associations of vitamin D concentration with metabolic and hormonal indices in women with polycystic ovary syndrome presenting abdominal and gynoidal type of obesity. Ginekol Pol.

[35] Bellan M (2014). Altered glucose metabolism rather than naive type 2 diabetes mellitus (T2DM) is related to vitamin D status in severe obesity. Cardiovasc Diabetol.

[36] Bril F (2015). Relationship of vitamin D with insulin resistance and disease severity in non-alcoholic steatohepatitis. J Hepatol.

[37] Lu Z (2015). Serum vitamin D levels are inversely related with non-alcoholic fatty liver disease independent of visceral obesity in Chinese postmenopausal women. Clin Exp Pharmacol Physiol.

[38] Ter Horst KW (2016). The vitamin D metabolites 25(OH)D and 1,25(OH)(2)D are not related to either glucose metabolism or insulin action in obese women. Diabetes Metab.

[39] Mousa A (2017). 25-hydroxyvitamin D is associated with adiposity and cardiometabolic risk factors in a predominantly vitamin D-deficient and overweight/obese but otherwise healthy cohort. J Steroid Biochem Mol Biol.

[40] Piantanida E (2017). Cardiometabolic healthy and unhealthy obesity: does vitamin D play a role?. Endocr Connect.

[41] Ong MW, Tan CH, Cheng AKS (2018). prevalence and determinants of Vitamin D deficiency among the overweight and obese Singaporeans seeking weight management including bariatric surgery: a relationship with bone health. Obes Surg.

[42] Curvello-Silva KL (2020). Association between cardiovascular risk factors and 25(OH)D levels in obese patients. Metab Syndr Relat Disord.

[43] Setayesh L, et al. Elevated plasma concentrations of vitamin D-binding protein are associated with lower high-density lipoprotein and higher fat mass index in overweight and obese women. Nutrients. 2021;13(9):3223. 10.3390/nu13093223 PMC8472481 34579103

[44] Gong T (2022). Vitamin D is negatively associated with triglyceride in overweight/obese patients with type 2 diabetes. Endocrine.

[45] Ruiz-Ojeda FJ (2018). Genetic factors and molecular mechanisms of Vitamin D and obesity relationship. Ann Nutr Metab.

[46] Abbas MA (2017). Physiological functions of Vitamin D in adipose tissue. J Steroid Biochem Mol Biol.

[47] Migliaccio S (2019). Obesity and hypovitaminosis D: causality or casualty?. Int J Obes Suppl.

[48] Wakeman M (2021). A literature review of the potential impact of medication on Vitamin D status. Risk Manag Healthc Policy.

[49] Webb AR (2010). The role of sunlight exposure in determining the vitamin D status of the U.K. white adult population. Br J Dermatol.

[50] Rodopaios NE (2021). Vitamin D status, vitamin D intake, and sunlight exposure in adults adhering or not to periodic religious fasting for decades. Int J Food Sci Nutr.

[51] Zemel MB (2002). Regulation of adiposity and obesity risk by dietary calcium: mechanisms and implications. J Am Coll Nutr.

[52] Duncan RE (2007). Regulation of lipolysis in adipocytes. Annu Rev Nutr.

[53] Chiu KC (2004). Hypovitaminosis D is associated with insulin resistance and beta cell dysfunction. Am J Clin Nutr.

[54] Asano L (2017). Vitamin D Metabolite, 25-hydroxyvitamin D, regulates lipid metabolism by inducing degradation of SREBP/SCAP. Cell Chem Biol.

[55] Querfeld U (1999). Antagonistic effects of vitamin D and parathyroid hormone on lipoprotein lipase in cultured adipocytes. J Am Soc Nephrol.

[56] Clemente-Postigo M (2011). Adipose tissue gene expression of factors related to lipid processing in obesity. PLoS ONE.

[57] Capell WH (1996). Compositional differences of LDL particles in normal subjects with LDL subclass phenotype A and LDL subclass phenotype B. Arterioscler Thromb Vasc Biol.

[58] Deeb SS (2003). Hepatic lipase and dyslipidemia: interactions among genetic variants, obesity, gender, and diet. J Lipid Res.

[59] Silverberg SJ (2007). Vitamin D deficiency and primary hyperparathyroidism. J Bone Miner Res.

[60] Procopio M (2014). Cardiovascular risk and metabolic syndrome in primary hyperparathyroidism and their correlation to different clinical forms. Endocrine.

