# Supplementary material for: Association between vitamin D deficiency and lipid profiles in overweight and obese adults: a systematic review and meta-analysis
Source: BMC Public Health. 2023 Aug 29;23:1653. doi: 10.1186/s12889-023-16447-4 (PMC10464009; doi:10.1186/s12889-023-16447-4)
Supplement: Supplementary file 2 — Additional file 2. [file 12889_2023_16447_MOESM2_ESM.docx]

**Supplementary Table 1: Definition of vitamin D deficiency of the included studies**

| No | Author,year | Definition of vitamin D deficiency |
| --- | --- | --- |
| 1 | Carretero 2007 | vitamin D insufficiency as those levels between 20 and 32 ng/ml (50 and 80 nmol/l, respectively), and vitamin D deficiency as those below 20 ng/ml (50 nmol/l). |
| 2 | Yildizhan2009 | ND |
| 3 | Muscogiuri 2010 | 25(OH)D concentration with a cutoff value of <50 nmol/l used to define low 25(OH)D concentration |
| 4 | Bellia 2011 | tertile 35.1–60.2 nmol/L |
| 5 | Esteghamati 2014 | A cut-off point of 20 ng/mL was used to categorise participants as either vitamin-D-deficient or non-deficient |
| 6 | boonchaya-anant2014 | Vitamin D deficiency was defined as serum 25(OH)D levels < 20 ng/ml and vitamin D insufficiency as 25(OH)D level of 21-29 ng/ml according to the Endocrine Society clinical practice guideline. 25(OH)D level of 30 ng/ml or above was considered to be vitamin D sufficiency. |
| 7 | Jarosław 2014 | Levels of 25(OH)D were interpreted as follows: ≥30 ng/mL: norm; 21-29 ng/mL: insufficiency; 11-20 ng/mL: deficiency; ≤10 ng/ml: deep deficiency. |
| 8 | Amena Sadiya 2014 | vitamin D status was recorded based on serum concentration of 25(OH)D as severely deficient (<25 nmol/L), deficient (<50 nmol/L), insufficient (<75 nmol/L), or normal (>75 nmol/L) |
| 9 | Bellan 2014 | 5(OH)D3 deficiency/insufficiency were set at 20 and 30 ng/ml, respectively |
| 10 | Bril 2015 | 25-hydroxyvitamin D levels (normal: >30 ng/ml; insufficiency: 20–30 ng/ml; deficiency: <20 ng/ml) |
| 11 | lu2015 | ND |
| 12 | Terhorst 2016 | 25(OH)D levels < 50 nmol/L (< 20 ng/mL) |
| 13 | mousa2016 | vitamin D deficient (25(OH)D≤50 nmol/L), 11% (n=12) as sufficient (25(OH)D >50 to ≤75 nmol/L), and replete (25(OH)D>75 nmol/L) |
| 14 | Piantanida 2017 | Vitamin D status was categorized as ‘severe deficiency’ (S-DEFD) <10 ng/mL 25(OH)D, ‘deficiency’ (DEFD)10–19.9 ng/mL, ‘insufficiency’ (INSUFD) 20–29.9 ng/mL and ‘sufficiency’ (SUFD) ≥30 ng/mL |
| 15 | ong2018 | Deficiency was defined as < 20 ng/mL, insufficiency as 20–30 ng/mL and sufficiency as > 30 ng/mL based on the US Endocrine Society Clinical Practice guidelines |
| 16 | Karine 2020 | 25(OH)D levels < 22 ng/dL) |
| 17 | Setayesh 2021 | The vitamin deficiency was defined at a serum concentration of vitamin D < 20 ng/ml (<50 nmoL/L), insufficiency in the range of 21–29 ng/mL (52.5–72.5 nmoL/L), and normal ≥ 30 ng/mL (≥75 nmoL/L) |
| 18 | Minna F. Schleu 2021 | vitamin D deficient (25(OH)D level: >20 or ≤30 ng/mL), insufficient (25(OH)D level: <20 ng/mL) and normal (25(OH)D level: >30 ng/mL) |
| 19 | Lara A da C. Dominoni 2022 | adequate ( ≥30 ng/mL), insufficient ( ≥20 and < 30 ng/mL), and deficient ( < 20 ng/mL) |
| 20 | Salah Gariballa 2022 | deficiency (<20 ng/mL), insufficiency (20-32ng/mL) or optimal (>32 ng/mL) |
| 21 | Tong Gong 2022 | ND |

ND: Not Determined;

|  | **Supplementary Table 2: Absolute values of lipid levels in vitamin D deficiency.** | | | | | | | | | | | | |
| --- | --- | --- | --- | --- | --- | --- | --- | --- | --- | --- | --- | --- | --- |
| TG | number | study | year | case_n | case_mean (mg/dl) | case_SD (mg/dl) | control_n | control_mean (mg/dl) | control_SD (mg/dl) | subgroup | BMI | age | VD |
|  | 1 | Carretero | 2007 | 24 | 163.3 | 81.5 | 20 | 95.1 | 24.2 | Male/Female | ＞35 | ＜40 | ＜50 |
|  | 2 | Muscogiuri | 2010 | 21 | 100.5 | 46.5 | 18 | 71.1 | 26.3 | Male/Female | 25-35 | 40-60 | ＜50 |
|  | 3 | Bellia | 2011 | 49 | 126.7 | 72.8 | 49 | 117 | 69.6 | Male/Female | ＞35 | ＜40 | ＜60.2 |
|  | 4 | Amena Sadiya | 2014 | 132 | 168.3 | 88.6 | 38 | 132.9 | 53.2 | Male/Female | ＞35 | 40-60 | ＜25 |
|  | 5 | bellan | 2014 | 444 | 133 | 53.5 | 25 | 130 | 49.6 | Male/Female | ＞35 | 40-60 | ＜50 |
|  | 6 | Bril | 2015 | 113 | 153 | 79.3 | 52 | 145 | 70.4 | Male/Female | ＞25-35 | 40-60 | ＜50 |
|  | 7 | Terhorst | 2016 | 16 | 94.5 | 59.1 | 21 | 79.7 | 26.3 | Female | ＞35 | 40-60 | ＜50 |
|  | 8 | Piantanida | 2017 | 60 | 142 | 74 | 40 | 134 | 59 | Male/Female | ＞35 | 40-60 | ＜25 |
|  | 9 | Karine | 2020 | 98 | 113 | 247.4 | 103 | 136 | 448.9 | Male/Female | ＞35 | ＜40 | ＜22 |
|  | 10 | Setayesh | 2021 | 74 | 114.85 | 62.42 | 162 | 120.89 | 75.15 | Female | 25-35 | ＜40 | ＜75 |
|  | 11 | Minna F. Schleu | 2021 | 27 | 134 | 58.5 | 76 | 124 | 56.3 | Female | ＞35 | 40-60 | ＜50 |
|  | 12 | Salah Gariballa | 2022 | 61 | 117.8 | 70 | 66 | 148 | 132.9 | Male/Female | 25-35 | 40-60 | ＜39 |
|  | 13 | Tong Gong | 2022 | 33 | 178.1 | 133.2 | 53 | 115.2 | 69.6 | Male/Female | 25-35 | 40-60 | ＜31.5 |
| TC | number | study | year | case_n | case_mean (mg/dl) | case_SD (mg/dl) | control_n | control_mean (mg/dl) | control_SD (mg/dl) | subgroup | BMI | age | VD |
|  | 1 | Carretero | 2007 | 24 | 188.5 | 40.2 | 20 | 178.4 | 24 | Male/Female | ＞35 | ＜40 | ＜50 |
|  | 2 | Muscogiuri | 2010 | 21 | 201.8 | 45.6 | 18 | 160.1 | 45.3 | Male/Female | 25-35 | 40-60 | ＜50 |
|  | 3 | Bellia | 2011 | 49 | 182.3 | 47.6 | 49 | 194.7 | 64.6 | Male/Female | ＞35 | ＜40 | ＜60.2 |
|  | 4 | Amena Sadiya | 2014 | 132 | 185.8 | 42.6 | 38 | 158.7 | 34.8 | Male/Female | ＞35 | 40-60 | ＜25 |
|  | 5 | bellan | 2014 | 444 | 192 | 36.4 | 25 | 200 | 48.7 | Male/Female | ＞35 | 40-60 | ＜50 |
|  | 6 | Bril | 2015 | 113 | 175 | 42.5 | 52 | 164 | 43.3 | Male/Female | ＞25-35 | 40-60 | ＜50 |
|  | 7 | Terhorst | 2016 | 16 | 185.8 | 42.6 | 21 | 174.2 | 27.1 | Female | ＞35 | 40-60 | ＜50 |
|  | 8 | Piantanida | 2017 | 60 | 202 | 36 | 40 | 213 | 32 | Male/Female | ＞35 | 40-60 | ＜25 |
|  | 9 | Karine | 2020 | 98 | 204.3 | 45.5 | 103 | 189.7 | 35.2 | Male/Female | ＞35 | ＜40 | ＜22 |
|  | 10 | Setayesh | 2021 | 74 | 186.6 | 35.29 | 162 | 184.14 | 36.52 | Female | 25-35 | ＜40 | ＜75 |
|  | 11 | Minna F. Schleu | 2021 | 27 | 209 | 49.6 | 76 | 190.5 | 32.6 | Female | ＞35 | 40-60 | ＜50 |
|  | 12 | Salah Gariballa | 2022 | 61 | 186.5 | 38.7 | 66 | 185 | 36.4 | Male/Female | 25-35 | 40-60 | ＜39 |
|  | 13 | Tong Gong | 2022 | 33 | 185.8 | 44.5 | 53 | 169.1 | 36 | Male/Female | 25-35 | 40-60 | ＜31.5 |
| LDL | number | study | year | case_n | case_mean (mg/dl) | case_SD (mg/dl) | control_n | control_mean (mg/dl) | control_SD (mg/dl) | subgroup | BMI | age | VD |
|  | 1 | Carretero | 2007 | 24 | 117.3 | 38.1 | 20 | 115 | 17.1 | Male/Female | ＞35 | ＜40 | ＜50 |
|  | 2 | Muscogiuri | 2010 | 21 | 120.8 | 44.5 | 18 | 90.3 | 36.6 | Male/Female | 25-35 | 40-60 | ＜50 |
|  | 3 | Amena Sadiya | 2014 | 132 | 116.1 | 38.7 | 38 | 92.9 | 27.1 | Male/Female | ＞35 | 40-60 | ＜25 |
|  | 4 | bellan | 2014 | 444 | 122 | 31.1 | 25 | 126 | 35 | Male/Female | ＞35 | 40-60 | ＜50 |
|  | 5 | Bril | 2015 | 113 | 100 | 31.9 | 52 | 94 | 36.1 | Male/Female | ＞25-35 | 40-60 | ＜50 |
|  | 6 | Terhorst | 2016 | 16 | 120 | 38.7 | 21 | 112.2 | 27.1 | Female | ＞35 | 40-60 | ＜50 |
|  | 7 | Piantanida | 2017 | 60 | 123 | 32 | 40 | 131 | 31 | Male/Female | ＞35 | 40-60 | ＜25 |
|  | 8 | Karine | 2020 | 98 | 118 | 170.9 | 103 | 112.6 | 91 | Male/Female | ＞35 | ＜40 | ＜22 |
|  | 9 | Setayesh | 2021 | 74 | 94.18 | 26.01 | 162 | 93.15 | 22.64 | Female | 25-35 | ＜40 | ＜75 |
|  | 10 | Minna F. Schleu | 2021 | 27 | 126.2 | 43 | 76 | 122.4 | 27.3 | Female | ＞35 | 40-60 | ＜50 |
|  | 11 | Salah Gariballa | 2022 | 61 | 126 | 36.8 | 66 | 122.2 | 31.7 | Male/Female | 25-35 | 40-60 | ＜39 |
|  | 12 | Tong Gong | 2022 | 33 | 185.8 | 44.5 | 53 | 169.1 | 36 | Male/Female | 25-35 | 40-60 | ＜31.5 |
| HDL | number | study | year | case_n | case_mean (mg/dl) | case_SD (mg/dl) | control_n | control_mean (mg/dl) | control_SD (mg/dl) | subgroup | BMI | age | VD |
|  | 1 | Carretero | 2007 | 24 | 37 | 7.8 | 20 | 44.9 | 8.7 | Male/Female | BMI＞35kg/m^2^ | ＜40 | ＜50 |
|  | 2 | Muscogiuri | 2010 | 21 | 56.3 | 14.5 | 18 | 52.7 | 12.5 | Male/Female | BMI＜35kg/m^2^ | 40-60 | ＜50 |
|  | 3 | Bellia | 2011 | 49 | 40.6 | 10.4 | 49 | 44.9 | 10.1 | Male/Female | BMI＞35kg/m^2^ | ＜40 | ＜60.2 |
|  | 4 | Amena Sadiya | 2014 | 132 | 42.6 | 11.6 | 38 | 46.4 | 11.6 | Male/Female | BMI＞35kg/m^2^ | 40-60 | ＜25 |
|  | 5 | bellan | 2014 | 444 | 41.5 | 11.3 | 25 | 41.5 | 16.7 | Male/Female | BMI＞35kg/m^2^ | 40-60 | ＜50 |
|  | 6 | Bril | 2015 | 113 | 38 | 10.6 | 52 | 40 | 7.2 | Male/Female | BMI＜35kg/m^2^ | 40-60 | ＜50 |
|  | 7 | Terhorst | 2016 | 16 | 38.7 | 7.7 | 21 | 46.4 | 11.6 | Female | BMI＞35kg/m^2^ | 40-60 | ＜50 |
|  | 8 | Piantanida | 2017 | 60 | 51 | 12 | 40 | 53 | 12 | Male/Female | BMI＞35kg/m^2^ | 40-60 | ＜25 |
|  | 9 | Karine | 2020 | 98 | 46 | 48.9 | 103 | 46 | 73.3 | Male/Female | BMI＞35kg/m^2^ | ＜40 | ＜22 |
|  | 10 | Setayesh | 2021 | 74 | 47.09 | 12 | 162 | 46.02 | 10.81 | Female | BMI＜35kg/m^2^ | ＜40 | ＜75 |
|  | 11 | Minna F. Schleu | 2021 | 27 | 39 | 9.6 | 76 | 42 | 7.4 | Female | BMI＞35kg/m^2^ | 40-60 | ＜50 |
|  | 12 | Salah Gariballa | 2022 | 61 | 48.4 | 14.3 | 66 | 47.2 | 13.9 | Male/Female | BMI＜35kg/m^2^ | 40-60 | ＜39 |
|  | 13 | Tong Gong | 2022 | 33 | 37.5 | 12.8 | 53 | 43 | 10.8 | Male/Female | BMI＜35kg/m^2^ | 40-60 | ＜31.5 |

VD: vitamin D; TG: triglycerides; TC: total cholesterol; LDL: low-density lipoprotein cholesterol; HDL: high-density lipoprotein cholesterol;

**Supplementary Table 3: Subgroup analyses of vitamin D deficiency on TG level.**

|  | WMD[95%CI] | P within  group | Heterogeneity | | | P of subgroup Meta-regression |
| --- | --- | --- | --- | --- | --- | --- |
|  |  |  | P heterogeneity | I^2^ | P between  Sub-groups |  |
| Overall effect | 15.01[2.51, 27.52] | 0.019 | 0.002 | 61.2% |  |  |
| Gender |  |  |  |  |  |  |
| Male/Female | 18.75[2.87, 34.63] | 0.021 | 0.002 | 64.9% | 0.121 | 0.410 |
| Female | 2.31[-11.11, 15.72] | 0.736 | 0.413 | 0.0% |  |  |
| Age(year) |  |  |  |  |  |  |
| ＜40 | 17.30[-19.52, 54.12] | 0.357 | 0.002 | 79.2% | 0.884 | 0.888 |
| ＞40 | 14.41[1.64, 27.18] | 0.027 | 0.040 | 50.6% |  |  |
| BMI(kg/m^2^) |  |  |  |  |  |  |
| ＜35 | 9.86[-13.51, 33.22] | 0.408 | 0.006 | 72.3% | 0.532 | 0.536 |
| ＞35 | 18.67[3.95, 33.39] | 0.013 | 0.042 | 52.0% |  |  |
| Publication year |  |  |  |  |  |  |
| Before 2015 | 23.64[6.59, 40.69] | 0.007 | 0.015 | 64.7% | 0.123 | 0.195 |
| After 2016 | 5.04[-11.35, 21.43] | 0.547 | 0.087 | 45.7% |  |  |
| Definition of vitamin D deficiency(nmol/l) |  |  |  |  |  |  |
| ＜(22-49) | 14.44[-16.01, 44.90] | 0.353 | 0.010 | 70.0% | 0.993 | 0.983 |
| ＜(50-75) | 14.60[0.90, 28.29] | 0.037 | 0.017 | 59.1% |  |  |

WMD: weighted mean difference;

**Supplementary Table 4: Subgroup analyses of vitamin D deficiency on TC level.**

|  | WMD[95%CI] | P within  group | Heterogeneity | | | P of subgroup Meta-regression |
| --- | --- | --- | --- | --- | --- | --- |
|  |  |  | P heterogeneity | I^2^ | P between  Sub-groups |  |
| Overall effect | 8.61[1.31, 15.92] | 0.021 | 0.001 | 63.1% |  |  |
| Gender |  |  |  |  |  |  |
| Male/Female | 8.46[-0.86, 17.77] | 0.075 | 0.000 | 70.1% | 0.808 | 0.914 |
| Female | 6.84[-2.31, 15.99] | 0.143 | 0.334 | 8.8% |  |  |
| Age(year) |  |  |  |  |  |  |
| ＜40 | 5.63[-4.07, 15.33] | 0.255 | 0.140 | 45.2% | 0.484 | 0.530 |
| ＞40 | 10.71[0.34, 21.07] | 0.043 | 0.001 | 69.9% |  |  |
| BMI(kg/m^2^) |  |  |  |  |  |  |
| ＜35 | 10.13[0.26, 20.00] | 0.044 | 0.073 | 53.2% | 0.664 | 0.614 |
| ＞35 | 6.85[-4.18, 17.88] | 0.224 | 0.001 | 70.7% |  |  |
| Publication year |  |  |  |  |  |  |
| Before 2015 | 11.12[-3.09, 25.33] | 0.125 | 0.003 | 71.9% | 0.568 | 0.604 |
| After 2016 | 6.39[-1.48, 14.26] | 0.111 | 0.055 | 51.4% |  |  |
| Definition of vitamin D deficiency(nmol/l) |  |  |  |  |  |  |
| ＜(22-49) | 9.65[-3.40, 22.70] | 0.147 | 0.001 | 78.4% | 0.798 | 0.840 |
| ＜(50-75) | 7.59[-1.35, 16.53] | 0.096 | 0.063 | 47.8% |  |  |

WMD: weighted mean difference;

**Supplementary Table 5: Subgroup analyses of vitamin D deficiency on LDL level.**

|  | WMD[95%CI] | P within  group | Heterogeneity | | | P of subgroup Meta-regression |
| --- | --- | --- | --- | --- | --- | --- |
|  |  |  | P heterogeneity | I^2^ | P between  Sub-groups |  |
| Overall effect | 6.12[0.02, 12.23] | 0.049 | 0.015 | 53.2% |  |  |
| Gender |  |  |  |  |  |  |
| Male/Female | 7.43[-0.92, 15.77] | 0.081 | 0.006 | 62.6% | 0.295 | 0.591 |
| Female | 1.89[-4.25, 8.04] | 0.545 | 0.828 | 0.0% |  |  |
| Age(year) |  |  |  |  |  |  |
| ＜40 | 1.32[-4.96, 7.61] | 0.680 | 0.969 | 0.0% | 0.223 | 0.473 |
| ＞40 | 7.67[-0.38, 15.73] | 0.062 | 62.3% | 0.007 |  |  |
| BMI(kg/m^2^) |  |  |  |  |  |  |
| ＜35 | 6.89[-0.50, 14.27] | 0.068 | 0.146 | 41.3% | 0.705 | 0.596 |
| ＞35 | 4.42[-5.97, 14.81] | 0.404 | 0.011 | 63.9% |  |  |
| Publication year |  |  |  |  |  |  |
| Before 2015 | 10.42[-1.45, 22.29] | 0.085 | 0.010 | 69.8% | 0.190 | 0.265 |
| After 2016 | 1.86[-2.93, 6.65] | 0.449 | 0.483 | 0.0% |  |  |
| Definition of vitamin D deficiency(nmol/l) |  |  |  |  |  |  |
| ＜(22-49) | 8.46[-4.76, 21.68] | 0.210 | 0.005 | 73.4% | 0.454 | 0.557 |
| ＜(50-75) | 3.07[-1.88, 8.01] | 0.224 | 0.400 | 3.4% |  |  |

WMD: weighted mean difference;

**Supplementary Table 6: Subgroup analyses of vitamin D deficiency on HDL level.**

|  | WMD[95%CI] | P within  group | Heterogeneity | | | P of subgroup Meta-regression |
| --- | --- | --- | --- | --- | --- | --- |
|  |  |  | P heterogeneity | I^2^ | P between  Sub-groups |  |
| Overall effect | -2.57[-4.26, -0.88] | 0.003 | 0.078 | 38.3% |  |  |
| Gender |  |  |  |  |  |  |
| Male/Female | -2.77[-4.57, -0.97] | 0.003 | 0.223 | 23.9% | 0.950 | 0.811 |
| Female | -2.61[-7.20, 1.98] | 0.265 | 0.033 | 70.7% |  |  |
| Age(year) |  |  |  |  |  |  |
| ＜40 | -3.20[-7.95, 1.55] | 0.186 | 0.017 | 70.6% | 0.764 | 0.776 |
| ＞40 | -2.43[-4.11, -0.75] | 0.005 | 0.323 | 13.4% |  |  |
| BMI(kg/m^2^) |  |  |  |  |  |  |
| ＜35 | -0.72[-3.23, 1.78] | 0.571 | 0.147 | 41.1% | 0.035 | 0.045 |
| ＞35 | -4.03[-5.80, -2.25] | 0.000 | 0.505 | 0.0% |  |  |
| Publication year |  |  |  |  |  |  |
| Before 2015 | -3.14[-5.51, -0.76] | 0.010 | 0.160 | 37.0% | 0.551 | 0.561 |
| After 2016 | -2.09[-4.59, 0.41] | 0.102 | 0.114 | 41.5% |  |  |
| Definition of vitamin D deficiency(nmol/l) |  |  |  |  |  |  |
| ＜(22-49) | -2.50[-4.84, -0.15] | 0.037 | 0.416 | 0.0% | 0.916 | 0.917 |
| ＜(50-75) | -2.68[-5.05, -0.30] | 0.027 | 0.030 | 54.9% |  |  |

WMD: weighted mean difference;
