# Supplementary material for: Association between vitamin D deficiency and lipid profiles in overweight and obese adults: a systematic review and meta-analysis
Source: BMC Public Health. 2023 Aug 29;23:1653. doi: 10.1186/s12889-023-16447-4 (PMC10464009; doi:10.1186/s12889-023-16447-4)
Supplement: Supplementary file 3 — Additional file 3. [file 12889_2023_16447_MOESM3_ESM.pptx]

## Slide 1
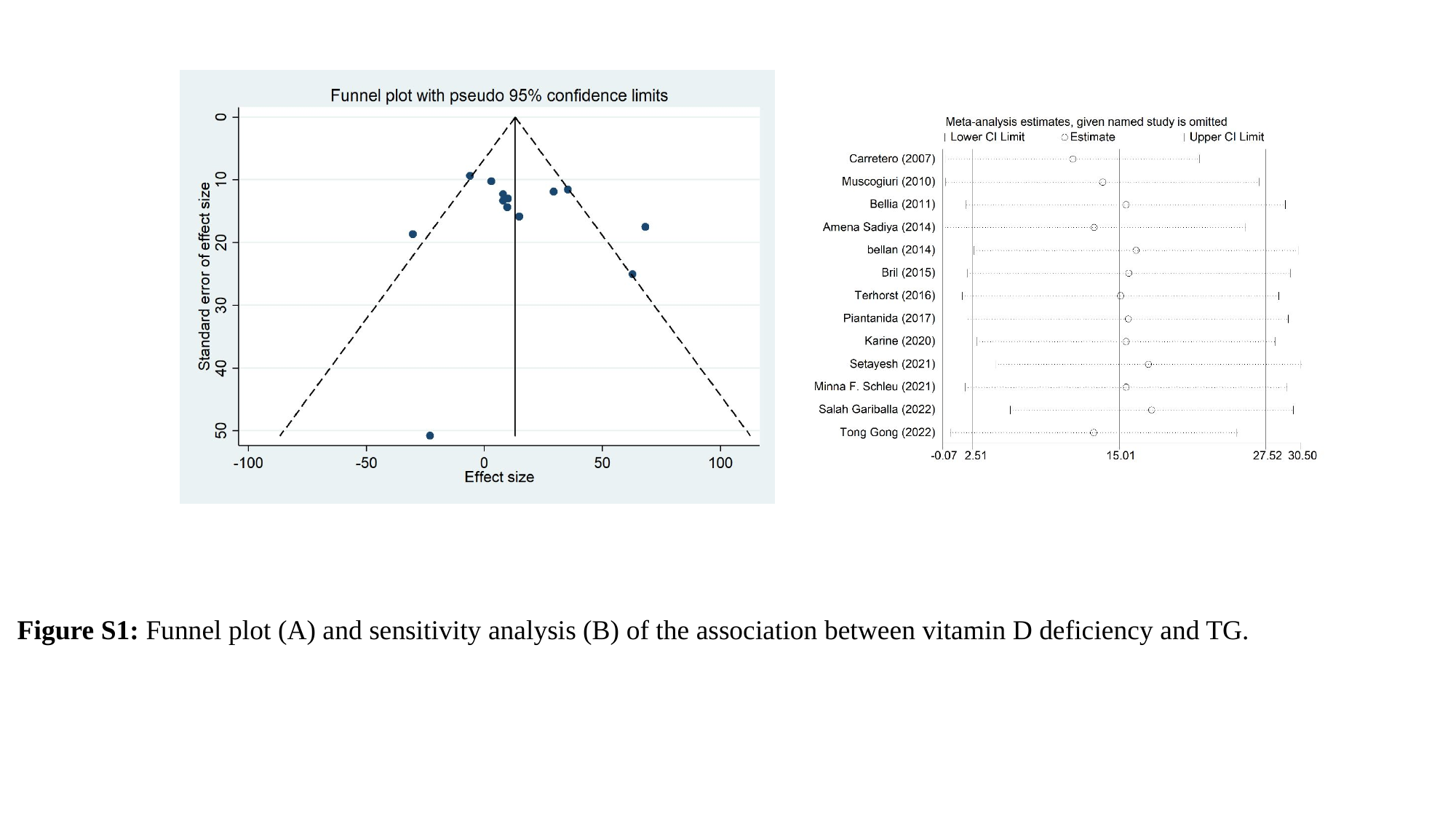

Figure S1: Funnel plot (A) and sensitivity analysis (B) of the association between vitamin D deficiency and TG.

## Slide 2
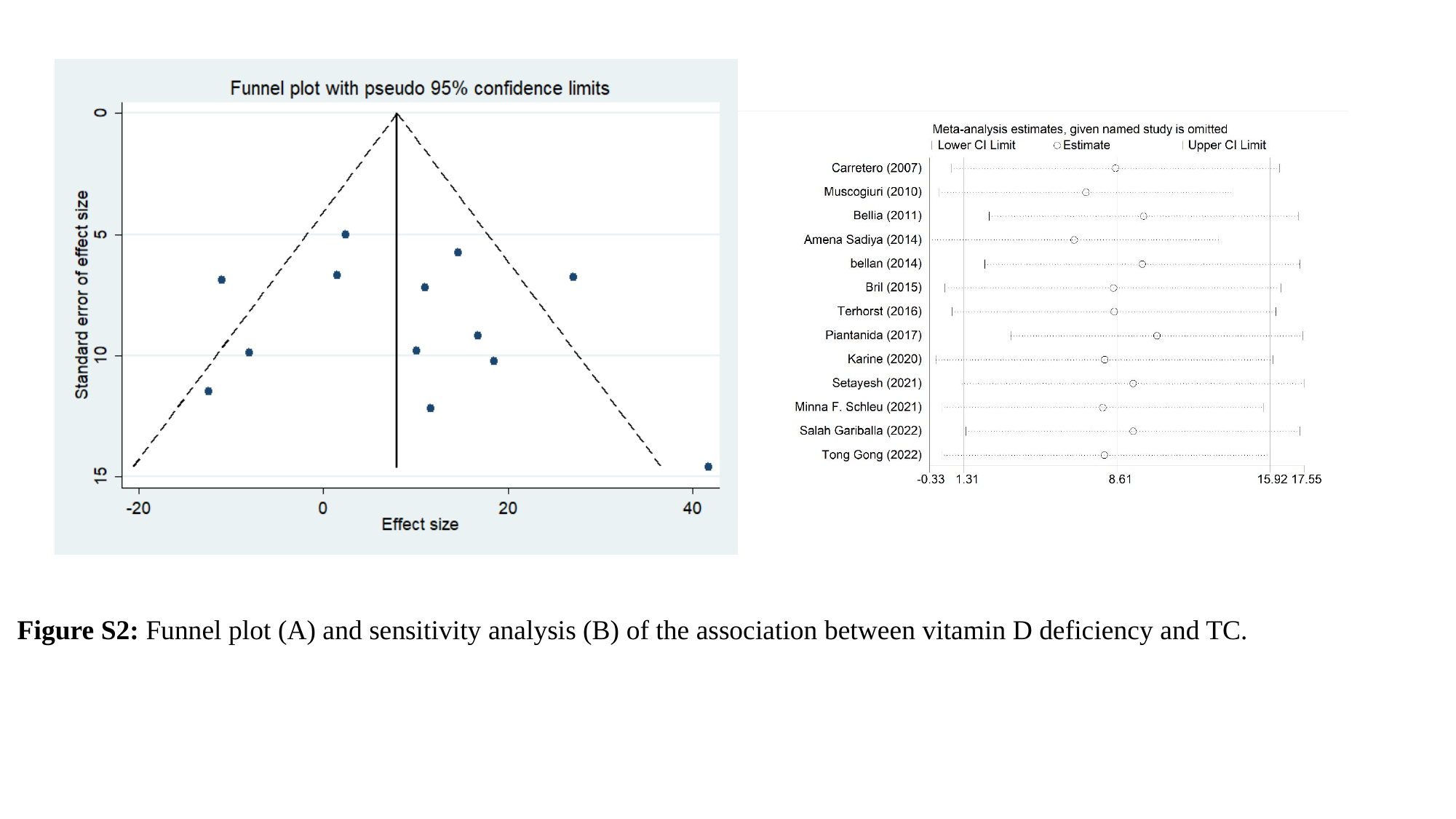

Figure S2: Funnel plot (A) and sensitivity analysis (B) of the association between vitamin D deficiency and TC.

## Slide 3
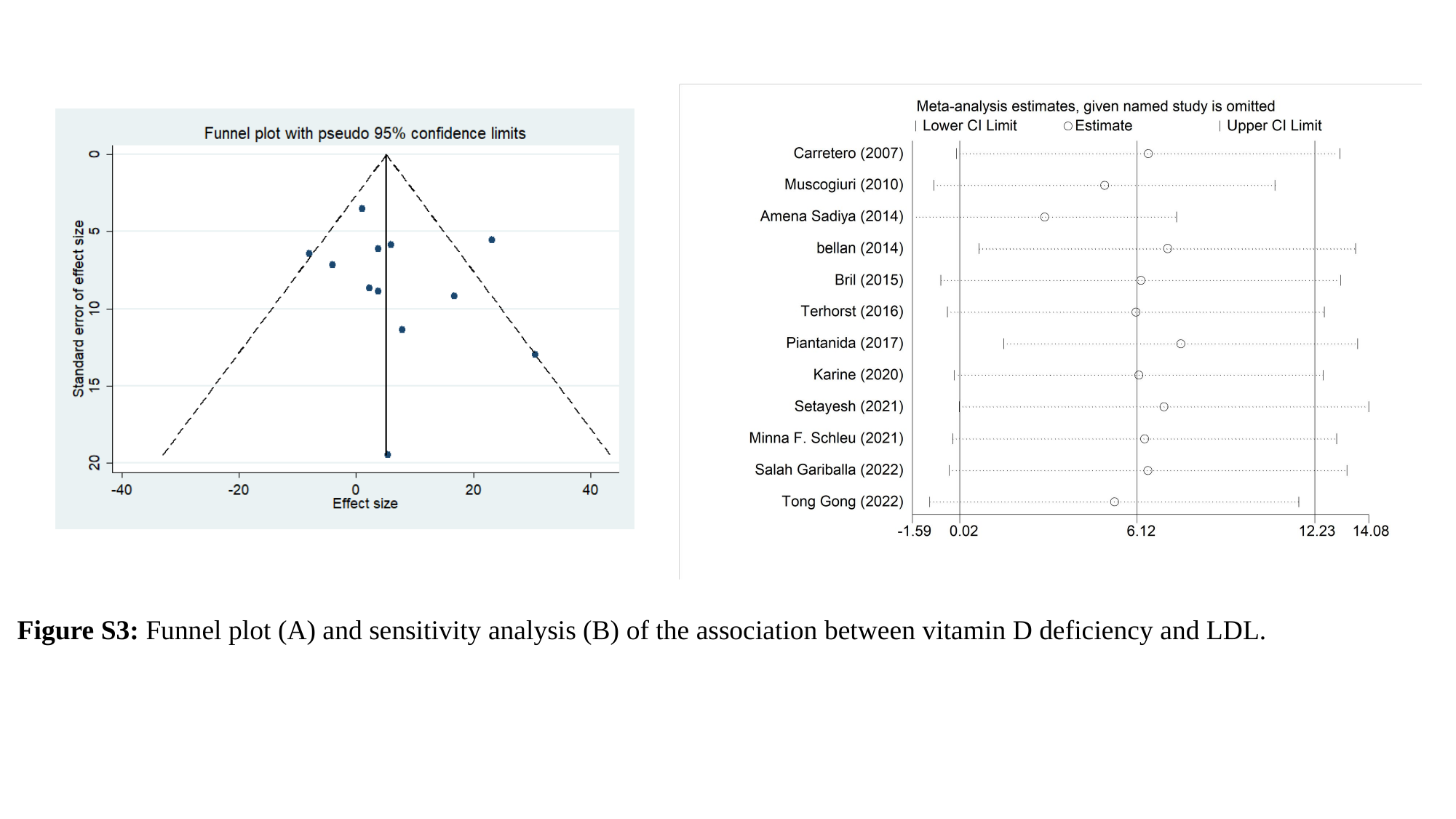

Figure S3: Funnel plot (A) and sensitivity analysis (B) of the association between vitamin D deficiency and LDL.

## Slide 4
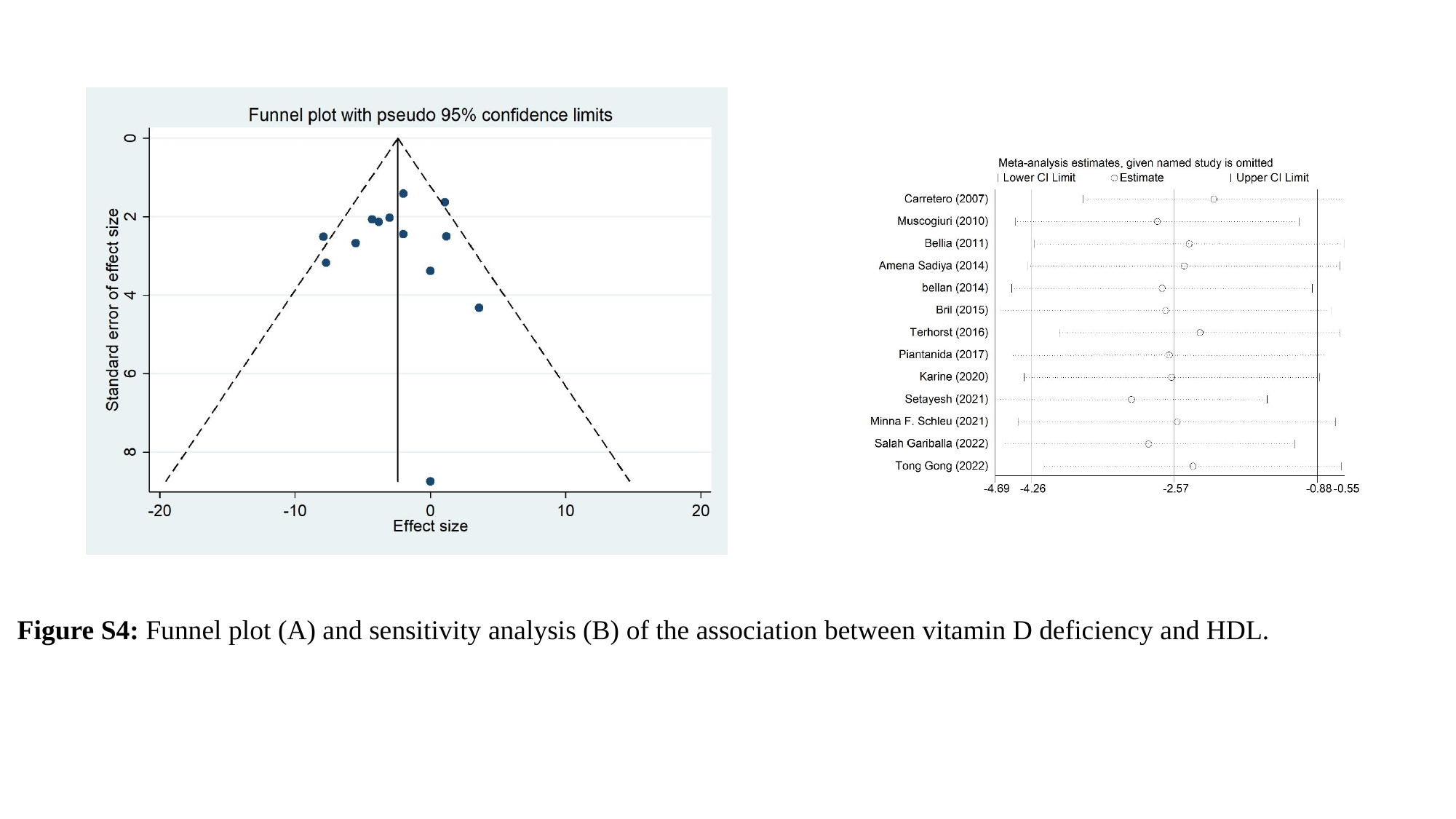

Figure S4: Funnel plot (A) and sensitivity analysis (B) of the association between vitamin D deficiency and HDL.
